# Reply to comment on “Ultrasonographic scores for ileal Crohn’s disease assessment: better, worse or the same as contrast‑enhanced ultrasound?

**DOI:** 10.1186/s12876-023-02883-4

**Published:** 2023-08-05

**Authors:** Marta Freitas, Francisca Dias de Castro, Vítor Macedo Silva, Cátia Arieira, Tiago Cúrdia Gonçalves, Sílvia Leite, Maria João Moreira, José Cotter

**Affiliations:** 1 Gastroenterology Department, Hospital da Senhora da Oliveira, Guimarães, Portugal; 2 Life and Health Sciences Research Institute (ICVS), School of Medicine, University of Minho, Braga, Portugal; 3 ICVS/3B’s, PT Government Associate Laboratory, Braga/Guimarães, Portugal

## Abstract

We read the comments by Nylund K et al. regarding our paper “Ultrasonographic scores for ileal Crohn’s disease assessment: Better, worse or the same as contrast‑enhanced ultrasound?”. Intestinal ultrasound has become one of the most valuable developments in the past decade, a non-invasive, well-tolerated exam, with an easy repeatability, and absence of sedation, ionizing radiation, or preparation. Particularly for inflammatory bowel disease, where there is a lack of agreement of patient’s symptoms with disease activity, in an era where the paradigm of mucosal healing is changing to transmural healing, and with the emergence of several therapies leading to repeated imaging surveillance, it is essential to highlight the role of intestinal ultrasound. Although intestinal ultrasound is an increasingly used tool to monitor inflammatory bowel disease activity, there is no widely accepted reproducible activity index, since the methodology for the development of the scores was shown to be insufficient in most studies and none have been adequately validated (Bots et al., J Crohns Colitis 12:920–9, 2018). In our study, we showed that the contrast-enhanced ultrasound (CEUS) peak enhancement derived from the time-intensity curve (TIC) is a promising non-invasive emerging method with a good accuracy to correlate clinical and endoscopic activity in the terminal ileum, superior to intestinal ultrasound scores relying on bowel wall thickness and colour Doppler.

We read the comments by Nylund K et al. [1] regarding our paper “Ultrasonographic scores for ileal Crohn’s disease assessment: Better, worse or the same as contrast‑enhanced ultrasound?”. In fact, our study aimed to compare, in our population, the accuracy of two intestinal ultrasound scores (SUS-CD score [2] and IBUS-SAS score [3]) and CEUS in predicting inflammatory activity in the terminal ileum in ileocolonoscopy in patients with Crohn’s disease, and not creating a score.

As pointed out by the authors, we recognize that the single-center, retrospective design of our study, and the selected population are limitations. We only included patients having an intestinal ultrasound, CEUS, and endoscopy, within 1 month, explaining the small sample of the study. Although we recognize that in clinical practice this may not occur in all the situations, we only included patients with these characteristics in order to limit potential bias regarding inflammatory activity variation. Regarding the reasons for which the assessment was performed we consider that this does not influence significantly the data as we are correlating endoscopic inflammatory activity with the ultrasound findings in the same subject.

Due to the retrospective design of our study we cannot use the concept “blinded” regarding the ultrasound operator to the results of the ileocolonoscopy. However, as mentioned in the text, we guarantee that the ultrasound operator was not the same that performed ileocolonoscopy examinations and the ileocolonoscopy examinations were performed after ultrasonographic examinations by an operator that does not have experience in ultrasound. Nevertheless, we recognize that, although the operator does not perform ultrasound, as a certified gastroenterologist he is able to interpret ultrasound findings.

Regarding the SES-CD threshold used in our study for inflammation, we highlight that there is no validated optimal SES-CD cut-off score and the quantification of disease severity has likewise not been standardized yet [4]. In our study, we graded the disease activity as inactive (normal or mild disease, with a SES-CD < 7) or active (moderate or severe disease, with a SES-CD ≥ 7), based on a previous study [5]. Several studies also considered this cut-off [6–9]. Although, some recent papers have used SES-CD with lower cut-offs, so we admit that the cut-off that we used (SES-CD ≥ 7) could be relatively high, and a lower value might have also been justified.

The authors highlighted that CEUS bowel wall perfusion measurements for detection of inflammation is only standard of care in select centers worldwide. However, in our center, we routinely perform CEUS in Crohn’s disease patients submitted to intestinal ultrasound as a part of a regular follow-up, including suspicion of active disease, assessment of remission or relapse, and monitoring of treatment effect. We recognize that it is a recent and promising tool, that needs more investigation, and that can contribute in clinical practice to the improvement of IBD activity monitoring. Evidence for the routine use of CEUS is based on small study groups and with significant methodological heterogeneity [10], with different ultrasound scanner and CEUS quantitative software, being the major issue for standardization, so there is still a need for large prospective studies that would help introducing the method into everyday practice.

In our paper a TIC curve based on wash-in wash-out is shown, not the fitted curve and the peak intensity was manually calculated.

Finally, we did not analyze inter-rater reliability as we only included ultrasonographic examinations performed by a single expert operator to uniformize the analysis. However, we agree that this is a major point for CEUS quantitative measurements, that needs to be addressed in future studies in order to demonstrate the reproducibility, and more objective character of CEUS.

## Data Availability

Not applicable.
